# Metastatic liver tumor from a primary gastric cancer with infiltration of the Glisson’s pedicle: A case report

**DOI:** 10.1016/j.ijscr.2019.05.012

**Published:** 2019-05-11

**Authors:** Yusuke Takahashi, Hitoshi Seki

**Affiliations:** Department of Digestive Surgery, Nagano Municipal Hospital, Nagano, 381-8551, Japan

**Keywords:** MR, magnetic resonance, PET, positron emission tomography, TNM, tumor-node-metastasis, CEA, carcinoembryonic antigen, CA, carbohydrate antigen, CT, computed tomography, Gd-EOB-DTPA, gadolinium-ethoxybenzyl-diethylenetriamine pentaacetic acid-enhanced, CDX, caudal-type homeobox, CK, cytokeratin, Stomach neoplasm, Liver neoplasms, Hepatectomy

## Abstract

**Introduction:**

Metastatic liver tumors from primary gastric cancer are rarely resected because of the high rate of metastasis and recurrence of gastric cancer, and there is little information regarding the pathological assessment of these tumors. We present a case of a single metastatic liver tumor from gastric cancer with invasion of the interlobular bile duct for which we achieved margin-free resection with good clinical outcomes.

**Presentation of case:**

An 80-year-old patient presented with a tumor in segment V of the liver, with invasion of Glisson’s pedicle confirmed on preoperative magnetic resonance and positron emission tomography imaging. On the basis of the preoperative assessment, we proceeded with partial hepatectomy, with transection of one of the roots of Glisson’s pedicle performed under echography guidance. Pathological examination confirmed gastric cancer as the primary source of the metastatic tumor. R0 resection was achieved, with no evidence of cancer recurrence at one year after surgery.

**Discussion:**

Our experience supports partial hepatectomy with R0 margin tumor resection as an oncologically feasible treatment for metastatic liver tumor arising from primary gastric cancer (in the absence of other metastatic lesions) and shows that a good prognosis can be achieved. Of note, intraoperative echography did not detect tumor invasion of Glisson’s pedicle, which was evident only on preoperative imaging.

**Conclusion:**

This case suggests that magnetic resonance and positron emission tomography imaging findings are important for surgical planning of hepatectomy. Further follow-up data and more cases are needed to completely define the clinical significance of tumor invasion of Glisson’s pedicle.

## Highlights

• A single metastatic liver tumour from a primary gastric cancer is rare.
• Glisson’s pedicle invasion was identified on preoperative MR and PET-CT imaging.
• Surgical resection, with R0 margin, was deemed possible based on imaging results.
• The patient survived, without cancer recurrence for more than 1 year.

## 1 Introduction

In carefully selected patients, a favorable prognosis is possible after resection of metastatic liver tumors arising from primary gastric cancer [1]. Partial (rather than anatomical) resection with an appropriate tumor-free (R0) margin is the basic therapeutic approach for any metastatic liver tumor, with intraoperative echography aiding in visualization of the tumor margins and surrounding vessels [2]. However, surgical resection is rarely indicated for metastatic liver tumors arising from primary gastric cancer because of the high incidence rate of metastasis (locally and within the peritoneal cavity) and high recurrence rate of this type of cancer [3]. Therefore, the clinicopathological features of liver metastases from primary gastric cancer and the surgical strategy for treatment remain to be clarified. Herein, we describe our diagnosis and surgical treatment of a patient with metastatic liver tumor from primary gastric cancer in whom magnetic resonance (MR) and positron emission tomography (PET) imaging indicated invasion of Glisson’s pedicle. On the basis of these images, we believed that we could achieve tumor resection with an R0 margin. Our case report adheres to the SCARE criteria [4].

## 2 Presentation of case

**Fig. 1:**
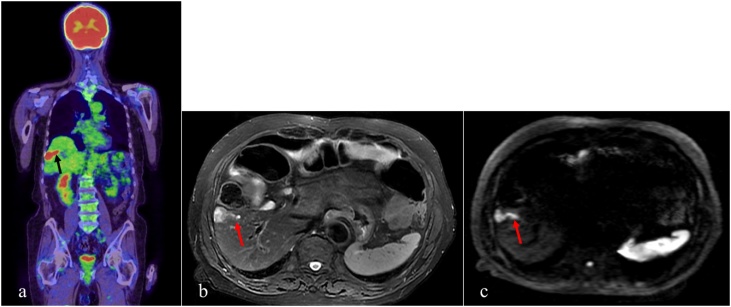
Preoperative images. a) Positron emission tomography image. b) Magnetic resonance (MR) T2-weighted image. c) MR imaging diffusion-weighted image. Glisson’s pedicle infiltration (arrow) was suspected on the basis of these images.

The patient was an 80-year-old man who had undergone distal gastrectomy for gastric cancer [T3N1M0 per tumor-node-metastasis (TNM) staging, 7^th^ classification] and colectomy for transverse colon cancer (T2N0M0 per TNM staging, 7^th^ classification) four years previously. Eight months after these surgeries, the patient underwent partial hepatectomy for a metastatic liver tumor arising from primary gastric cancer, which was pathologically confirmed. The patient refused to undergo adjuvant chemotherapy. Periodic assessment of tumor marker levels (carcinoembryonic antigen (CEA) and carbohydrate antigen 19-9 (CA 19-9)) and enhanced computed tomography (CT) were performed during follow-up. Enhanced CT performed 4 years after the gastric/colon surgeries revealed a new low-intensity mass, 16 mm in diameter, located in segment Ⅴ of the liver; the location of this lesion was different from that of the lesion identified during the first liver surgery. There was no evidence of other local, peritoneal, or lung metastases. On PET–CT, the mass presented as a hot, linear-like lesion, that almost appeared as two separate lesions (Fig. 1a). Consistent with enhanced CT examination, PET-CT examination did not reveal evidence of other metastatic lesions. T2- and diffusion-weighted images from gadolinium-ethoxybenzyl-diethylenetriamine pentaacetic acid-enhanced (Gd-EOB-DTPA) MR imaging revealed a high-intensity mass with invasion of Glisson’s pedicle in segment V of the liver (Fig. 1b/c). The CEA level was elevated, whereas the CA 19-9 level was within the normal range. Considering these clinical data, we suspected a metastatic liver tumor arising from either primary gastric or transverse colon cancer. As imaging studies indicated that liver resection and cholecystectomy could be achieved with no residual tumor, we proceeded with surgical treatment.

Although the mass and Glisson’s pedicle in segment V of the liver were visible on intraoperative echography, invasion of Glisson’s pedicle, identified on MR imaging and PET examinations, was unclear. Accordingly, following cholecystectomy, we proceeded with partial hepatectomy and transection of the root of Glisson’s pedicle in segment V, under intraoperative echography guidance.

**Fig. 2:**
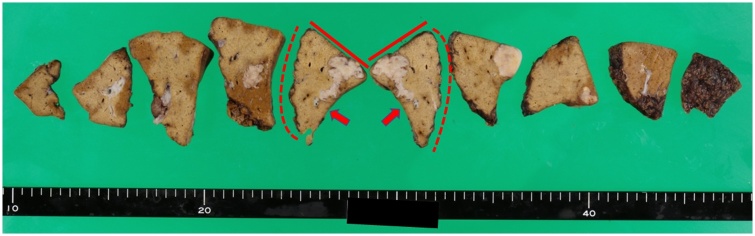
Cut surface of the resected liver. The straight line indicates the liver surface, the arrow indicates the gallbladder bed, and the dotted line indicates the cut surface.

**Fig. 3:**
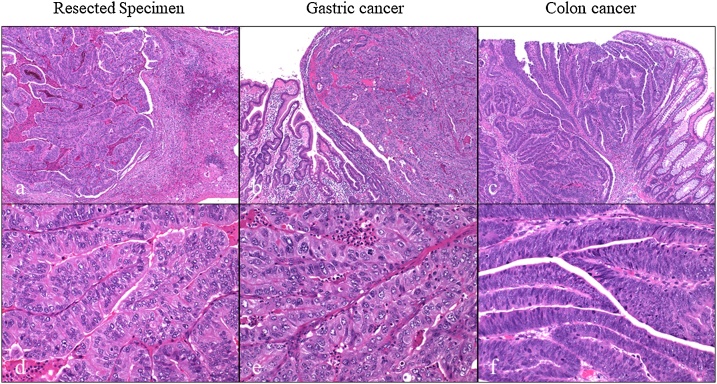
Pathological findings (Hematoxylin-eosin staining). a)–c) Magnification, ×40. d)–f) Magnification, ×200. The resected tumor was pathologically similar to the primary gastric cancer rather than colon cancer.

**Fig. 4:**
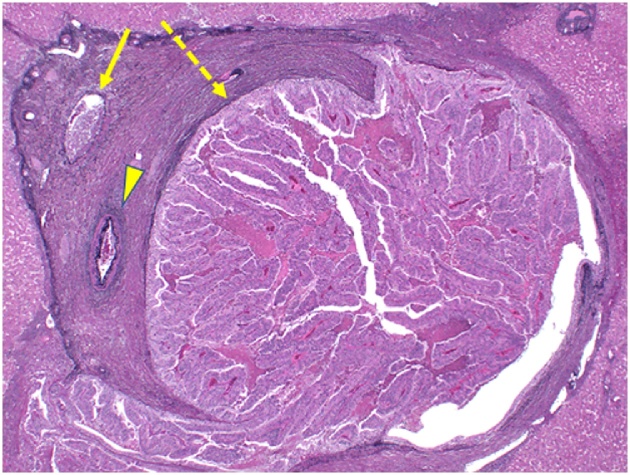
Elastica hematoxylin-eosin staining (magnification, ×20). The straight line indicates the interlobular vein, and the arrow head indicates the interlobular artery. The dotted line indicates metastatic infiltration of the interlobular bile duct.

**Fig. 5:**
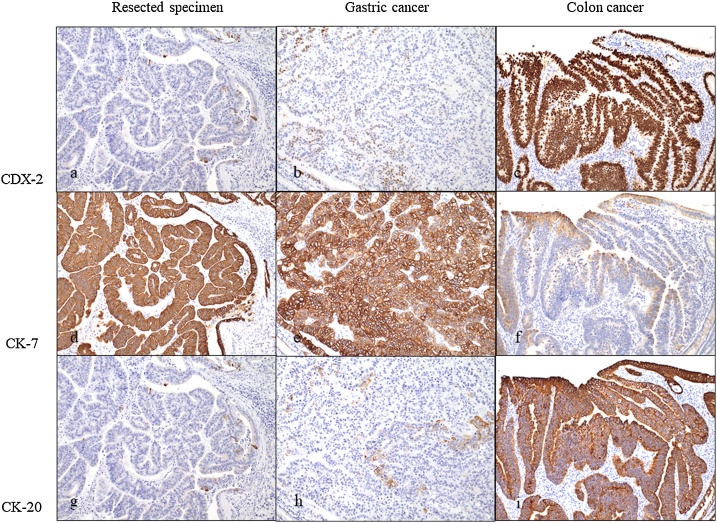
Immunohistochemical staining (magnification, ×100). a)–c) CDX-2 levels. The nuclei of the primary colon cancer cells were diffusely positive, while those of the metastatic and primary gastric cancer cells were negative. d)–f) CK-7 levels. The epithelium of the metastatic and primary gastric cancer samples was highly positive, and that of the primary colon cancer sample was weakly positive (judged as negative). g)–i) CK-20 levels. The epithelium of the colon cancer sample was diffusely and highly positive, while the epithelium was negative in the metastatic and primary gastric cancer samples. Immunohistochemical findings of the resected specimen were compatible with those of the primary gastric cancer, but not with those of the colon cancer. CDX, caudal-type homeobox; CK, cytokeratin.

On gross appearance, the surgical margin of the tumor was within the cut surface (Fig. 2). On the basis of the pathological reports of previously resected specimens of gastric and colon cancers, the resected tumor was pathologically similar to gastric cancer (Fig. 3). Tumor infiltration of Glisson’s pedicle, including the interlobular bile duct, was also confirmed on pathological analysis (Fig. 4). Immunohistochemical staining was negative for caudal-type homeobox (CDX)-2 and cytokeratin (CK)-20 and positive for CK-7; these findings were compatible with those of previous gastric cancer rather than colon cancer (Fig. 5). Thus, a final diagnosis of metastatic liver tumor from primary gastric cancer was made. One year after surgery, tumor marker (CEA and CA 19-9) levels remained within the normal range, and the patient was alive, with no evidence of recurrence.

## 3 Discussion

This is, to our knowledge, the first case report of invasion of Glisson’s pedicle by a metastatic liver tumor arising from primary gastric cancer, although cases of intrabiliary metastases from colorectal cancer have been reported [5,6]. Metachronous liver metastasis arising from gastric cancer, with no other metastatic lesions, is a rare occurrence, and in this case, a good prognosis was obtained. The surgical strategy for the treatment of metastatic liver tumors from primary gastric cancer is controversial, largely because of the overall poor prognosis of gastric cancer. Chemotherapy (using S-1 plus cisplatin) is the standard treatment for metastatic gastric cancer, with a median overall survival of 13 months, according to the results of the SPIRITS trial [7]. The occurrence of only liver metastasis from primary gastric cancer is rare because of the higher prevalence of peritoneal or local metastases and the high rate of gastric cancer recurrence [3]; thus, surgical resection for metastatic liver tumor from gastric cancer is rarely indicated. However, the findings of a multicenter retrospective analysis indicate a 5-year survival rate of approximately 30% for metastatic liver tumors from gastric cancer with surgical indication [8,9]. In our case, a single tumor in the liver was detected on PET and (Gd-EOB-DTPA)-MR examinations, with Glisson’s pedicle invasion, which was not detected on the enhanced CT examination. Despite the metastatic nature of the tumor, preoperative imaging indicated that R0 resection was possible. Thus, on the basis of the clinical findings, we proceeded with hepatectomy, and the patient remained without cancer recurrence for more than one year after surgery. Moreover, a good prognosis is expected on the basis of the patient’s survival to date, which at the time of publication was 42 and 34 months from the initial surgery (concurrent colectomy and gastrectomy) and first liver surgery (first partial hepatectomy), respectively.

Anatomical hepatectomy is the treatment of choice for primary liver tumors and hepatocellular carcinomas, which often infiltrate the portal vein. Partial hepatectomy is typically sufficient for the treatment of metastatic liver tumors; Glisson’s pedicle invasion is generally not observed in metastatic liver tumors, particularly small tumors [10]. In our case, as invasion of Glisson’s pedicle was suspected on preoperative images, anatomical hepatectomy of segment V (namely, subsegmentectomy) was initially planned. Of note, although invasion of Glisson’s pedicle was not observed on intraoperative echography, we proceeded with transection of one of the roots of Glisson’s pedicle under intraoperative echography guidance, on the basis of preoperative imaging examinations. Our procedure was classified as partial hepatectomy, rather than anatomical hepatectomy. (Gd-EOB-DTPA)-MR and PET-CT imaging were useful for surgical planning to achieve R0 resection in the current case.

Metastatic cancer cells may migrate with the bile flow to the distal bile duct, and recurrence in the hepatoduodenal ligament, peritoneal dissemination, or lymph node metastasis may occur. The clinical significance of Glisson’s pedicle invasion (particularly of the interlobular bile duct) with metastatic gastric tumor is, however, largely unknown because of the rarity of this condition. Considering the absence of recurrence in the remnant liver one year after surgery, including the remnant segment V, partial hepatectomy with R0 resection may be an oncologically acceptable option if invasion of Glisson’s pedicle is detected on preoperative imaging. Further postoperative follow-up would clarify the clinical significance of this type of cancer, considering the relatively short-term follow-up (one year from final surgery) in the current case.

## 4 Conclusion

We treated a patient with a metastatic liver tumor arising from gastric cancer, with infiltration of Glisson’s pedicle, including the interlobular bile duct. (Gd-EOB-DTPA)-MR (T2- and diffusion-weighted images) and PET-CT imaging were useful modalities for detecting the invasion of Glisson’s pedicle and for surgical planning. Considering the one-year survival of our patient with no cancer recurrence, partial resection with an R0 margin may be appropriate for oncologic treatment of metastatic liver tumors from gastric cancer with Glisson’s pedicle invasion in the absence of other metastatic lesions.

## Conflicts of interest

There are no conflicts of interest.

## Funding

This research did not receive any specific grant from funding agencies in the public, commercial, or not-for-profit sectors.

## Ethical approval

Ethical approval was not obtained for this case report.

## Consent

Written informed consent was obtained from the patients for publication of this case report and accompanying figures. A copy of the written consent is available for review by the Editor-in-Chief of this journal on request.

## Author contribution

Study concept, design, and writing of this case report were by author: Yusuke Takahashi. Hitoshi Seki participated in the treatment of the patient and drafted the manuscript. Hitoshi Seki critically revised the manuscript. All authors read and approved the final manuscript.

## Registration of Research Studies

This case report was not registered in a publicly accessible database.

## Guarantor

Yusuke Takahashi accepts full responsibility for the work and the conduct of the case report, had access to the data, and controlled the decision to publish.

## Provenance and peer review

Not commissioned, externally peer-reviewed.

## References

[1] Nishi M., Shimada M., Yoshikawa K., Higashijima J., Tokunaga T., Kashihara H. (2018). Results of hepatic resection for liver metastasis of gastric cancer—a single center experience. J. Med. Invest..

[2] Makuuchi M., Hasegawa H., Yamazaki S. (1983). Intraoperative ultrasonic examination for hepatectomy. Ultrasound Med. Biol..

[3] Nashimoto A., Akazawa K., Isobe Y., Miyashiro I., Katai H., Kodera Y. (2013). Gastric cancer treated in 2002 in Japan: 2009 annual report of the JGCA nationwide registry. Gastric Cancer.

[4] Agha R.A., Borrelli M.R., Farwana R., Koshy K., Fowler A., Orgill D.P., For the SCARE Group (2018). The SCARE 2018 statement: updating consensus surgical case report (SCARE) guidelines. Int. J. Surg..

[5] Onishi I., Kayahara M., Takei R., Makita N., Munemoto M., Yagi Y. (2018). Recurrent biliary dissemination of colon cancer liver metastasis: a case report. J. Med. Case Rep..

[6] Sasaki S., Nomura Y., Fukutomi S., Shirahama N., Kusano H., Akiba J. (2019). Intrabiliary growth type of metastasis from colon cancer, 12 years after curative colectomy: a case report. BMC Surg..

[7] Koizumi W., Narahara H., Hara T., Takagane A., Akiya T., Takagi M. (2008). S-1 plus cisplatin versus S-1 alone for first-line treatment of advanced gastric cancer (SPIRITS trial): a phase III trial. Lancet Oncol..

[8] Kinoshita T., Kinoshita T., Saiura A., Esaki M., Sakamoto H., Yamanaka T. (2015). Multicentre analysis of long-term outcome after surgical resection for gastric cancer liver metastases. Br. J. Surg..

[9] Oki E., Tokunaga S., Emi Y., Kusumoto T., Yamamoto M., Fukuzawa K. (2016). Surgical treatment of liver metastasis of gastric cancer: a retrospective multicenter cohort study (kscc1302). Gastric Cancer.

[10] Makuuchi M., Hasegawa H., Yamazaki S. (1985). Ultrasonically guided subsegmentectomy. Surg. Gynecol. Obstet..

